# Three new species of the genus *Scaphicoma* Motschulsky, 1863 (Coleoptera, Staphylinidae, Scaphidiinae) from Northern Sulawesi, Indonesia

**DOI:** 10.3897/zookeys.403.7200

**Published:** 2014-04-17

**Authors:** Ryo Ogawa, Ivan Löbl, Kaoru Maeto

**Affiliations:** 1 Laboratory of Insect Biodiversity and Ecosystem Science, Graduate School of Agricultural Science, Kobe University, 1-1 Rokkodai, Nada-ku, Kobe, 657-8501 Japan; 2 Muséum d’histoire naturelle, Route de Malagnou 1, CH-1208 Geneva, Switzerland

**Keywords:** Scaphidiinae, *Scaphicoma*, new species, male and female genitalia, Sulawesi, Indonesia, Sundaland

## Abstract

Three new species of the genus *Scaphicoma* Motschulsky, 1863 from Sulawesi, Indonesia are illustrated and described: *Scaphicoma subflava* Ogawa & Löbl, **sp. n.**, *S. bidentia* Ogawa & Löbl, **sp. n.**, and *S. quadrifasciata* Ogawa & Löbl, **sp. n.** *Lepteroscapha pallens* Achard, 1921 is designated as the type species of the genus *Lepteroscapha* Achard, 1921.

## Introduction

The genus *Scaphicoma* Motschulsky, 1863 currently includes 16 species (Achard 1920; Löbl 1971, 1973, 1984, 1990, 1992, 2003; Löbl and Leschen 2010; Ogawa and Hoshina 2012; Pic 1915, 1923), which are mainly distributed in Southeast Asia, with the exception of *Scaphicoma hiranoi* (Hoshina, 2008) from Japan, *Scaphicoma antennalis* (Achard, 1922) and *Scaphicoma yapo* Löbl & Leschen, 2010 from tropical Africa, and *Scaphicoma gracilis* Löbl, 1971 and *Scaphicoma montheisi* Löbl & Leschen, 2010 from New Ireland and Australia, respectively. This genus may be easily distinguished from other scaphidiines by body elongate, strongly convex in dorsal view, hind tarsus longer than hind tibia, mesepimera fused, and strongly elongate antennae.

In this paper, we describe three new species of the genus *Scaphicoma* from the northern and central parts of Sulawesi, Indonesia, and discuss the relationships among these new species and their relatives with respect to the geohistory of Sulawesi. In addition, we designate a type species of the genus *Lepteroscapha*.

## Material and methods

The specimens examined were collected by the first author or had been deposited at MHNG (see below). We refer to Ogawa and Löbl (2013) and the references quoted therein for methods and terminological conventions.

The abbreviations used herein are as follows:

EL length of elytra from base of pronotum to apex of elytra

EW maximum width of elytra

HW maximum width of head including eye

ID interocular distance

PL maximum length of pronotum

PW maximum width of pronotum

EUMJ Ehime University Museum, Matsuyama, Japan

HUOI Hasanuddin University, R. Ogawa collection, Makassar, Indonesia

MZBI Museum Zoologicum Bogorience, Bogor, Indonesia

MHNG Muséum d’histoire naturelle, Genève, Switzerland

RISTEK Ministry of State for Research and Technology, Indonesia

## Systematics

### Scaphicoma

Motschulsky, 1863

http://species-id.net/wiki/Scaphicoma

[gender: feminine]

Scaphicoma Motschulsky, 1863: 435; type species: *Scaphicoma flavovittata* Motschulsky, 1863; by monotypy.
Lepteroscapha Achard, 1921: 88; type species: *Lepteroscapha pallens* Achard, 1921; by present designation. Synonymy: Löbl 1971.

#### Note.

Achard (1921) established *Lepteroscapha* for three new species, *Lepteroscapha pallens*, *Lepteroscapha nigrovittata*, and *Lepteroscapha filiformis*. A type species was not designated. Therefore, we designate here *Lepteroscapha pallens* Achard, 1921 as the type species.

#### Key to Sulawesian species of *Scaphicoma*

**Table:** 

1	Body unicolorous (Fig. 1a, b)	2
–	Body bicolorous (Fig. 1c). Ventral surface with iridescent luster due to microsculptures. Parameres enlarged subapically and tapering to apex, weakly pointed around subapical portion in dorsal view	*Scaphicoma quadrifasciata* sp. n.
2	Body yellowish-brown to reddish-brown. Ventral surface not iridescent. Body 2.55–2.75 mm long. Antennomere XI about 1.6 times as long as VIII; IV and V shorter than VI. Mesotarsomere I about 1.2 times as long as II and about 2.2 times as long as IV; V about 1.5 times as long as IV. Metatarsomeres I about 1.5 times as long as II; IV almost as V length. Male sternite VII with strongly concave middle of apical margin. Parameres asymmetrical. Bursa copulatrix sclerotized (Fig. 2c)	*Scaphicoma subflava* sp. n.
–	Body dark reddish-brown. Body 2.25–2.44 mm long. Antennomere XI about two times as long as VIII; IV and V almost same as VI. Mesotarsomere I about 1.5 times as long as II; V about 2 times as long as IV. Metatarsomere I about 2.0 times as long as II and about 2.5 times as long as IV; V about 0.7 times as long as IV. Male and female sternite VII with moderately concave middle of apical margin. Paramere symmetrical, weakly enlarged subapically. Bursa copulatrix not sclerotized (Fig. 3c)	*Scaphicoma bidentia* sp. n.

### Scaphicoma subflava

Ogawa & Löbl sp. n.

http://zoobank.org/5EF0186B-9ECA-4998-805B-BC38A0450214

http://species-id.net/wiki/Scaphicoma_subflava

Figs 1a , 2 , 5

#### Diagnosis.

Most of body yellowish-brown. Body size relatively moderate. Antennomere XI about 1.6 times as long as VIII; IV and V each shorter than VI. Protarsomeres I–III and V each about two times as long as IV. Mesotarsomeres I about 1.2 times as long as II and III; V about 1.5 times as long as IV. Metatarsomeres I about 1.5 times as long as II and III; II and III each about 1.5 times as long as IV and V. Male sternite VII with middle of apical margin strongly concave. Parameres asymmetrical. Internal sac on basal portion covered with scale-like sclerites, and with pair of sclerites on apical portion. Bursa copulatrix sclerotized.

#### Description.

**Figure 1.**
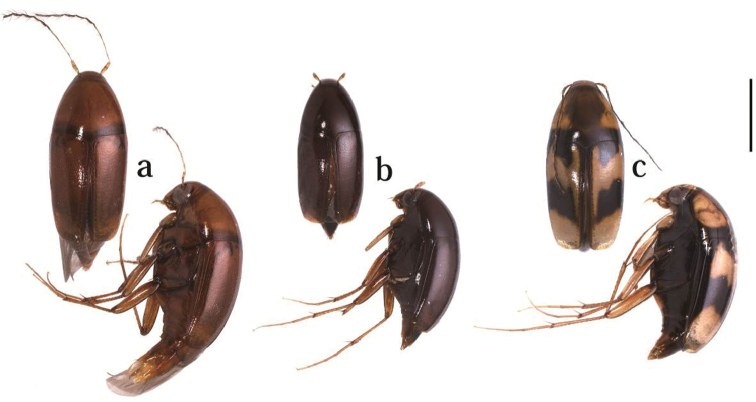
Habitus of *Scaphicoma* spp. in dorsal and lateral views. **a** *Scaphicoma subflava* sp. n. **b** *Scaphicoma bidentia* sp. n. **c** *Scaphicoma quadrifasciata* sp. n. Scale: 1.0 mm.

Body, shining. Most of body including head, pronotum and elytra yellowish-brown, except for darkened mesoventrite (Fig. 1a). Antennae yellowish-brown, except of antennomeres VII–XI dark yellowish-brown. Head, pronotum and elytra sparsely and finely pubescent.

**Figure 2.**
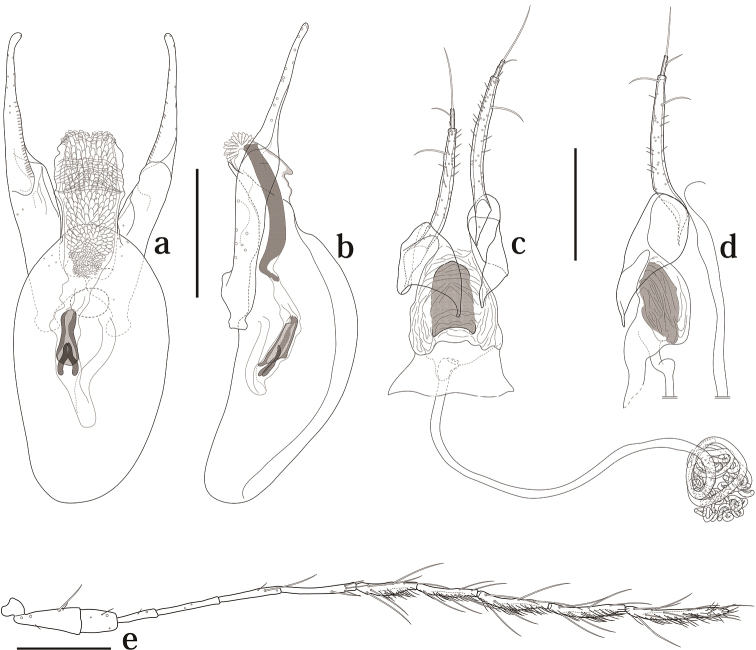
Genitalia and antenna of *Scaphicoma subflava* sp. n. **a, b** Male genitalia; **c, d** female genitalia **e** male antenna. a, c, Dorsal view; b, d, lateral view. Scale: 0.25 mm.

Head with interocular distance almost as eye width. Punctuation sparse and fine. Antennomeres I–VI with a few macrosetae, VII–XI covered with some microsetae; VI about two times as long as III; IV and V each shorter than VI; VII almost as VIII; XI about 1.6 times as long as VIII (Fig. 2e).

Pronotum almost as wide as long, lateral keel invisible in dorsal view. Punctuation sparse and fine, as on head. Scutellum concealed.

Elytra longer than wide, widest at basal 1/6, gradually narrowed to apex, with minute serration at inner part of posterior margin. Punctuation coarser than on pronotum (Fig. 5e, f). Sutural striae extending outwards along basal margin to form basal striae, reaching humeral area and not joined with lateral striae.

Propygidium densely and finely punctuate. Pygidium sparsely and finely punctuate, slightly emarginated at apex.

Hypomeron finely punctuate. Lateral portion of mesoventrite coarsely and sparsely punctuate; medial portion finely and sparsely punctuate, with fine pubescence. Lateral portion of metaventrite from base to basal 1/3 sparsely and coarsely punctuate, with apical portion moderately concave. Mesocoxa almost six times as wide as space between them; mesocoxal area moderately broadened. Metepimeron almost as long as wide, with microsculpture. Metacoxa about eight times as wide as metacoxal process. Metanepisternum about six times as long as wide. Lateral portion of ventrite I from base to basal 1/3 densely and coarsely punctuate. Ventrite VI strongly pointed at apicomedian portion.

Meso- and metafemora with microsculpture, sparsely and coarsely punctuate. Protarsomeres I–III and V each about two times as long as IV. Mesotarsomeres I about 1.2 times as long as II and III; V about 1.5 times as long as IV. Metatarsomeres I about 1.5 times as long as II and III; II and III each about 1. 5 times as long as IV and V.

Male. Ventrite V strongly emarginated at apex. Protarsomeres I–III with tenent setae (Fig. 5b), enlarged. Aedeagus about 0.91 mm long; parameres asymmetrical; internal sac on basal portion covered with scale-like sclerites, and with a pair of sclerites (Fig. 2a).

Female. Ventrite V slightly emarginate or truncate. Protarsomeres I–III without tenent setae, not enlarged. Gonostylus elongate. Distal gonocoxite normal and elongate; vagina membranous, without robust sclerites; bursa copulatrix strongly sclerotized (Fig. 2c, d). Spermatheca as Fig. 2c.

#### Measurements

(n = 6). Length (PL+EL): 2.55–2.75 mm; width (PW, EW): 1.04–1.13 mm, 1.09–1.21 mm. HW: 0.55–0.58 mm. ID: 0.16–0.19 mm. PL/PW: 0.95–1.07. EL/EW:1.30–1.43. Approximate ratio of each antennal length (width) from base to apex as follows (n = 1): 1.7 (0.7): 0.9 (0.6): 1.0 (0.2): 1.6 (0.2): 1.5 (0.2): 1.9 (0.2): 1.5 (0.3): 1.5 (0.2): 1.7 (0.3): 1.9 (0.3): 2.5 (0.2).

#### Specimens examined.

Holotype, 1 ♂, Mt. Tilongkabila (Gunung Tilongkabila), N. Sulawesi, alt. ca. 800m, 0°34'28.52N, 123°11'30.61E, 8. VI. 2012, R. Ogawa leg. (MZBI); Paratypes, 2♂2♀, Same data above (EUMJ); 1♂, Mt. Tilongkabila (Gunung Tilongkabila), N. Sulawesi, alt. ca. 800–1300 m, 0°34'28.52N, 0°35'18.14N, 123°11'30.61E, 123°13'22.71E, 9. VI. 2012, R. Ogawa leg. (HUOI); 1♂, Palu, Palopo, C. Sulawesi, 25–27. VIII. 1990, A. Riedel leg. (MHNG).

#### Distribution.

Indonesia: northern and central Sulawesi.

#### Etymology.

This specific name is the Latin *subflava* adjective meaning somewhat yellowish.

#### Remarks.

This species was illustrated in Leschen and Lobl (2005), thought unidentified. It is very similar to the Javanese *Scaphicoma pallens* (Achard, 1921) by the body color and the shapes of male genitalia, but it is easily distinguished by the distinctive male genitalia with internal sac bearing sclerites.

### Scaphicoma bidentia

Ogawa & Löbl sp. n.

http://zoobank.org/03705635-A4E6-4878-91A6-804E5EF413C8

http://species-id.net/wiki/Scaphicoma_bidentia

Figs 1b , 3

#### Diagnosis.

Body dark reddish-brown. Body size relatively small. Antennomere XI about two times as long as VIII; IV and V each almost as VI. Protarsomeres I–III each about 1.2 times as long as IV; V about 1.7 times as long as IV. Mesotarsomeres I and V each about two times as long as II; III about 1.5 times as long as IV. Metatarsomeres I about 2.0 times as long as II; II and III each about 1. 5 times as long as IV; V about 1.7 times as long as IV. Male and female sternite VII with middle of apical margin moderately concave. Paramere symmetrical, weakly enlarged at subapical portion. Internal sac with two-pronged spear shaped sclerite.

#### Description.

Body shining. Head and mouthparts reddish-brown. Antenna yellowish-brown, except for antennomeres VI–XI dark yellowish-brown. Pronotum, elytra and ventral surface dark reddish-brown (Fig. 1b). Legs, propygidium and pygidium yellowish-brown. Head, pronotum and elytra sparsely and finely pubescent.

**Figure 3.**
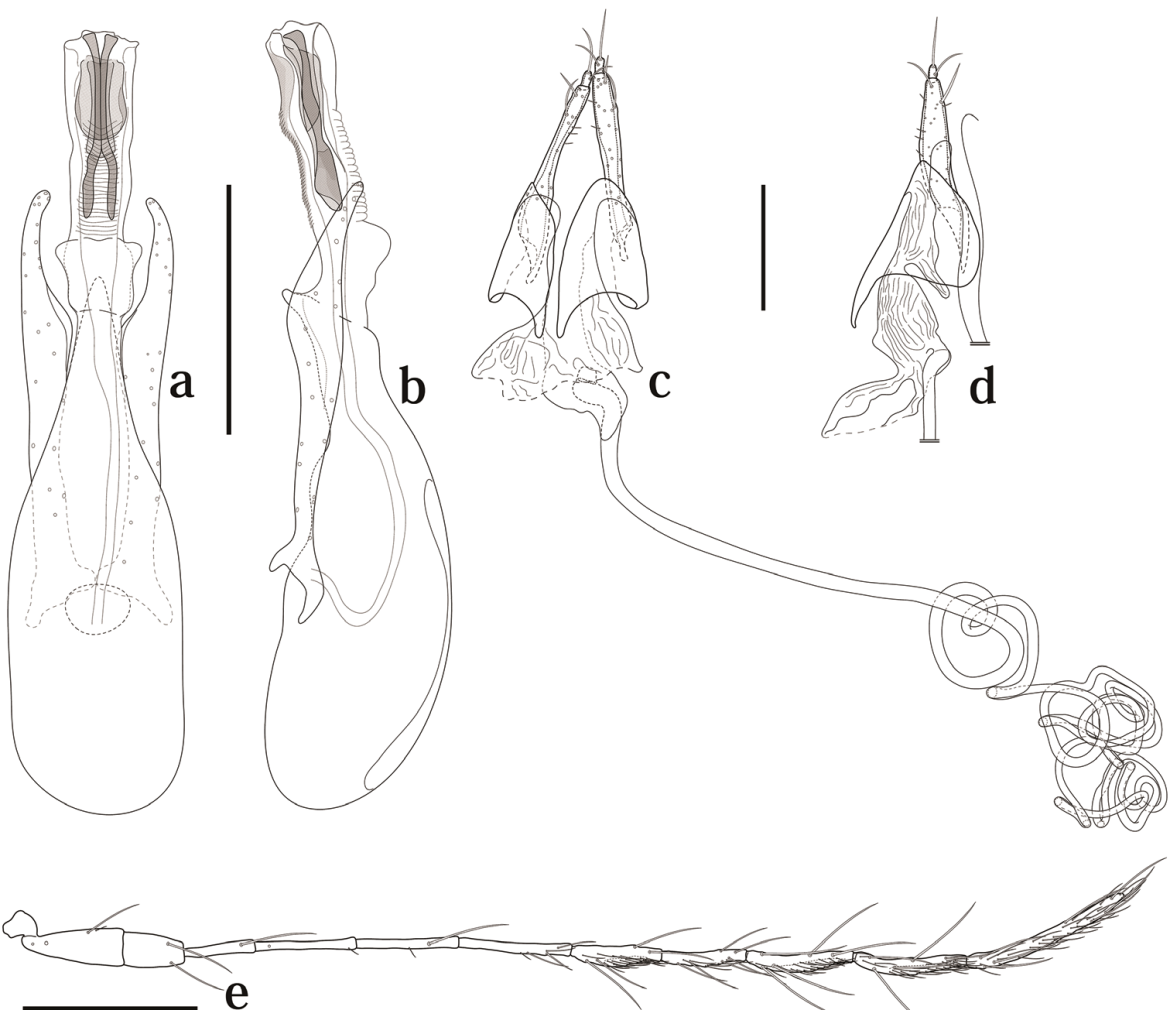
Genitalia and antenna of *Scaphicoma bidentia* sp. n. **a, b** Male genitalia **c, d** female genitalia **e** male antenna. **a, c** Dorsal view; **b, d** lateral view. Scale: 0.25 mm.

Head with interocular distance almost as eye width. Punctuation sparse and fine. Antennomeres I–VI with a few macrosetae, VII–XI covered with some microsetae; VI about 1.5 times as long as III; IV and V each almost as VI or shorter; VII almost as VIII or shorter; XI about two times as long as VIII (Fig. 3e).

Pronotum almost as wide as long, lateral keel invisible in dorsal view. Punctuation sparse and fine, as on head. Scutellum concealed.

Elytra longer than wide, widest at basal 1/6, gradually narrowed to apex, with minute serration at inner part of posterior margin. Punctuation coarser than on pronotum. Sutural striae extending outwards along basal margin to form basal striae, reaching humeral area and not joined with lateral striae.

Propygidium and pygidium sparsely and finely punctuate.

Hypomeron finely punctuate. Lateral portion of mesoventrite coarsely and sparsely punctuate; medial portion finely and sparsely punctuate, with fine pubescence. Lateral portion of metaventrite from base to basal 1/3 sparsely and coarsely punctuate, with apical portion moderately concave. Mesocoxa almost six times as wide as space between them; mesocoxal area moderately broadened. Metepimeron almost as long as wide, with microsculpture. Metacoxa about eight times as wide as metacoxal process. Metanepisternum about six times as long as wide. Lateral portion of ventrite I from base to basal 1/3 densely and coarsely punctuate. Ventrite V emarginated at apex. Ventrite VI strongly pointed at apicomedian portion.

Meso- and metafemora with microsculpture, sparsely and coarsely punctuate. Protarsomeres I–III each about 1.2 times as long as IV; V about 1.7 times as long as IV. Mesotarsomeres I and V each about two times as long as II; III about 1.5 times as long as IV. Metatarsomeres I about 2.0 times as long as II; II and III each about 1. 5 times as long as IV; V about 1.7 times as long as IV.

Male. Protarsomeres I–III with tenent setae, enlarged. Aedeagus about 0.62 mm long; parameres symmetrical; internal sac with two-pronged spear shaped sclerite (Fig. 3a).

Female. Protarsomeres I–III without tenent setae, not enlarged. Gonostylus robust. Distal gonocoxite normal, robust in lateral view; vagina membranous, without robust sclerites; bursa copulatrix not sclerotized (Fig. 3c, d). Spermatheca as Fig. 3c.

#### Measurements

(n = 3). Length (PL+EL): 2.25–2.44 mm; width (PW, EW): 0.89–0.94 mm, 0.93–0.98 mm. HW: 0.46–0.53 mm. ID: 0.16–0.19 mm. PL/PW: 1.00–1.20. EL/EW: 1.42–1.45. Approximate ratio of each antennal length (width) from base to apex as follows (n = 1): 1.4 (0.5): 0.9 (0.5): 1.0 (0.2): 1.4 (0.2): 1.4 (0.2): 1.6 (0.2): 1.3 (0.3): 1.2 (0.2): 1.5 (0.3): 1.6 (0.2): 2.4 (0.2).

#### Specimens examined.

Holotype, 1♂, Mt. Tilongkabila (Gunung Tilongkabila), N. Sulawesi, alt. ca. 500–800 m, 0°34'04.62N, 0°34'28.52N, 123°11'15.42E, 123°11'30.61E, 26–27. I. 2011, R. Ogawa leg. (MZBI); Paratypes, 1♀, same data above (EUMJ); 1♀, Mt. Tilongkabila (Gunung Tilongkabila), N. Sulawesi, alt. ca. 800–1300 m, 0°34'28.52N, 0°35'18.14N, 123°11'30.61E, 123°13'22.71E, 9.VI. 2012, R. Ogawa leg. (HUOI).

#### Distribution.

Indonesia: northern Sulawesi.

#### Etymology.

This specific name is derived from the Latin *bidentia* (two-pronged), referring to the shape of sclerites of the internal sac.

#### Remarks.

This species is very similar to the Philippines *Scaphicoma pullex* (Heller, 1917) by the body color and size, and it is also very similar to *Scaphicoma cincta* (Pic, 1920) from Sumatra by the shape of internal sac of the aedeagus. However, *Scaphicoma pullex* is easily distinguished from the new species by the Y-shaped sclerite of internal sac and *Scaphicoma cincta* is also easily distinguished from the new species by the color of elytra and pronotum with black along the edges.

### Scaphicoma quadrifasciata

Ogawa & Löbl sp. n.

http://zoobank.org/0450A718-3766-49A6-9D86-BAB2D5A5FC8E

http://species-id.net/wiki/Scaphicoma_quadrifasciata

Figs 1c , 4

#### Diagnosis.

Body bicolorous: basic color yellowish-brown, elytra each with black fasciae and black along sutural and lateral margins. Ventral surface with iridescent luster due to microsculptures. Antennomere VI about two times as long as III; IV and V each shorter than VI; XI about 1.6 times as long as VIII. Protarsomeres I–III and V about two times as long as IV. Mesotarsomeres I about 1.8 to 2.0 times as long as II; II, III and V each about 1.2 times as long as IV. Metatarsomeres I about 1.5 to 1.7 times as long as II; II and III each about 1.2 times as long as IV and V; IV almost as long as V. Parameres enlarged at subapical portion and tapering to apex, weakly pointed around subapical portion in dorsal view.

#### Description.

Body shining. Head, mouthparts and antenna yellowish-brown, except for antennomeres VII–XI dark yellowish-brown. Basic color of dorsal surface yellowish-brown, pronotum ochraceous or darkened on disc, black along margins. Elytra each with two black fasciae and black along suture and lateral margins (Fig. 1c). Posterior margins of anterior fasciae extended to apex along sutural striae, not reaching to sutural striae. Posterior fasciae extended to apex, reaching to sutural striae. Propygidium and pygidium from in basal half black, pygidium from mid-length to apex brown. Ventral surface almost black and with iridescent luster due to microsculptures. Coxa, ventrite I and II and femora, tibiae and tarsi yellowish-brown. Head, pronotum and elytra sparsely and finely pubescent.

**Figure 4.**
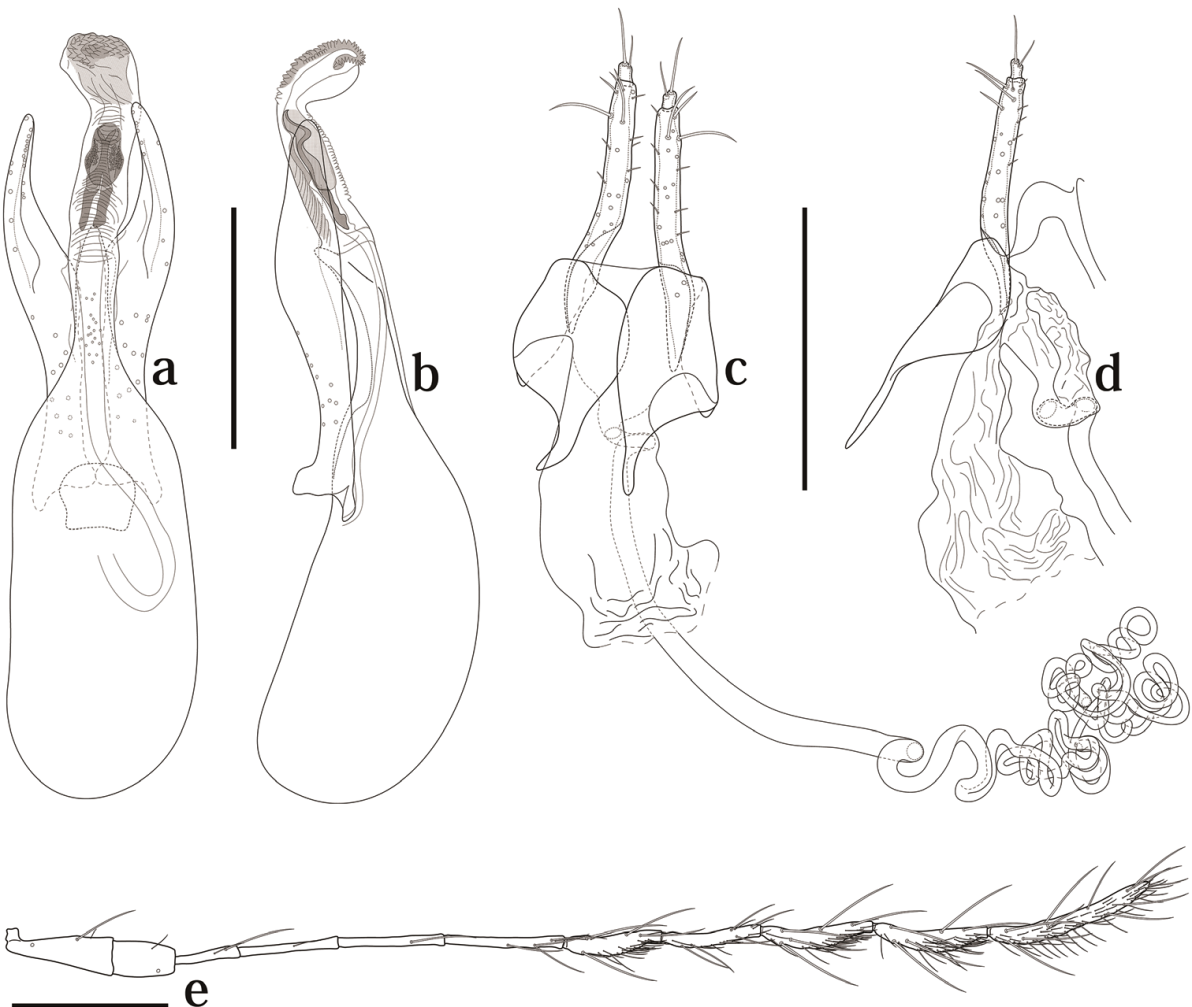
Genitalia and antenna of *Scaphicoma quadrifasciata* sp. n. **a, b** Male genitalia **c, d** female genitalia **e** female antenna. **a, c** Dorsal view; **b, d** lateral view. Scale: 0.25 mm.

Head with interocular distance almost as eye width. Punctuation sparse and fine. Antennomeres I–VI with a few macrosetae, VII–XI covered with some microsetae; VI about two times as long as III; IV and V each shorter than VI; VII almost as VIII; XI about 1.6 times as long as VIII (Fig. 4e).

**Figure 5.**
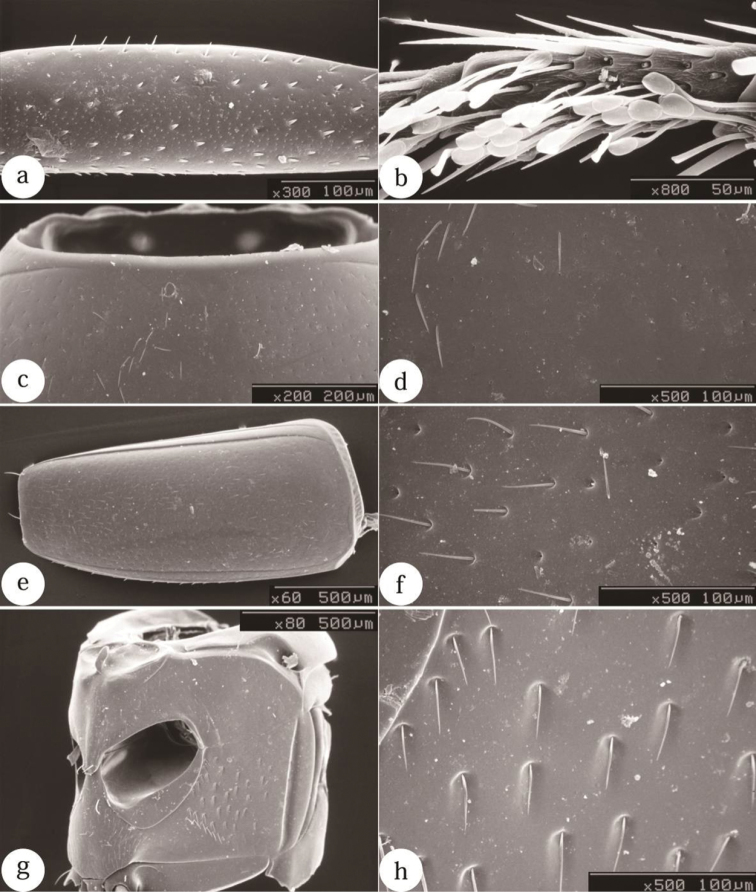
SEM photographs of a male of *Scaphicoma subflava* sp. n. **a** Profemur **b** tarsonomere III **c** anterior portion of pronotum **d** disc of pronotum **e** elytra **f** disc of elytra **g** meso- and metaventrite in oblique angle **h** lateral portion of metaventrite. **a, c–f** Dorsal view; **g, h** ventral view.

Pronotum almost as wide as long, lateral keel invisible in dorsal view. Punctuation sparse and fine, as on head. Scutellum concealed.

Elytra longer than wide, widest at basal 1/6, gradually narrowed to apex, with minute serration at inner part of posterior margin. Punctuation coarser than on pronotum. Sutural striae extending outwards along basal margin to form basal striae, reaching humeral area and not joined with lateral striae.

Propygidium sparsely and coarsely punctuate. Pygidium with sparse, fine and also coarse punctures.

Hypomeron finely punctuate. Lateral portion of mesoventrite coarsely and sparsely punctuate; medial portion finely and sparsely punctuate, with fine pubescence. Lateral portion of metaventrite from base to basal 1/3 sparsely and coarsely punctuate, with apical portion moderately concave. Mesocoxa almost six times as wide as space between them, mesocoxal area moderately broadened. Metanepisternum about six times as long as wide. Metepimeron almost as long as wide, with microsculptures. Metacoxa about eight times as wide as metacoxal process. Lateral portion of ventrite I from base to basal 1/3 densely and coarsely punctuate. Ventrite V moderately emarginated at apex. Ventrite VI strongly pointed at apical median portion.

Meso- and metafemora with microsculpture, sparsely and coarsely punctuate. Protarsomeres I–III and V each about two times as long as IV. Mesotarsomeres I about 1.8 to 2.0 times as long as II; II, III and V each about 1.2 times as long as IV. Metatarsomeres I about 1.5 to 1.7 times as long as II; II and III each about 1.2 times as long as IV and V; IV almost as long as V.

Male. Protarsomeres I–III with tenent setae, weakly enlarged. Aedeagus about 0.6 mm long; parameres symmetrical, enlarged at subapical portion, tapering to apex, weakly pointed around subapical portion in dorsal view; internal sac with two-pronged spear shaped sclerite, fine scale-like and denticulate structures (Fig. 4a).

Female. Protarsomeres I–III without tenent setae, not enlarged. Ovipositor simple; bursa copulatrix not sclerotized (Fig. 4c, d). Spermatheca as Fig. 4c.

#### Measurements

(n = 5). Length (PL+EL): 2.47–2.59 mm; width (PW, EW): 1.00–1.03 mm, 1.09–1.10 mm. HW: 0.51–0.54 mm. ID: 0.18–0.21 mm. PL/PW: 0.95–0.99. EL/EW: 1.36–1.46. Approximate ratio of each antennal length (width) from base to apex as follows (n = 1): 1.6 (0.6): 1.0 (0.6): 1.0 (0.2): 1.6 (0.2): 1.7 (0.2): 1.9 (0.2): 1.5 (0.3): 1.6 (0.2): 1.7 (0.3): 1.8 (0.3): 2.6 (0.3).

#### Specimens examined.

Holotype, 1♂, Mt. Tilongkabila (Gunung Tilongkabila), N. Sulawesi, alt. ca. 1300 m, 0°35'18.14N, 123°13'22.71E, 10. VI. 2012, R. Ogawa leg. (MZBI); Paratypes, 1♂1♀, Mt. Tilongkabila (Gunung Tilongkabila), N. Sulawesi, alt. ca. 800–1300 m, 0°34'28.52N, 0°35'18.14N, 123°11'30.61E, 123°13'22.71E, 9. VI. 2012, R. Ogawa leg. (EUMJ); 1♂1♀, same data above (HUOI).

#### Distribution.

Indonesia: northern Sulawesi.

#### Etymology.

This specific name is derived from the Latin *quadri* (four) and *fasciata* (band), referring to the four black elytral bands.

#### Remarks.

This species is very similar to *Scaphicoma nigrovittata* (Achard, 1921) and *Scaphicoma flavovittata* Motschulsky, 1863 from Sri Lanka by the distinctly bicolorous body. However, both may be distinguished from the new species by the almost black venter of body, the subapically enlarged parameres and by the shape of the sclerites of the internal sac.

## Discussion

Sulawesi is considered to have been formed by the collision of three continental plates, from which derive Sundaland (including Borneo, Sumatra and Java), the Philippines and Australia (e.g. Spakman and Hall 2010). Therefore, the fauna of northern Sulawesi is assumed to be associated with that of Sundaland and the Philippines (Michaux 2010, Stelbrink et al. 2012). Indeed, two Sulawesi species of beetle, *Scaphicoma subflava* and *Scaphicoma bidentia*, have closely related congeners in Java and the Philippines, respectively. In contrast, *Scaphicoma quadrifasciata* is probably related to congeners from Sri Lanka. Thus its ancestors may have drifted east-wards on ocean current. As there are still many gaps in our knowledge of *Scaphicoma*, further research is needed to gain a better understanding of the relationships and origins of the Sulawesi species.

## Supplementary Material

XML Treatment for Scaphicoma

XML Treatment for Scaphicoma subflava

XML Treatment for Scaphicoma bidentia

XML Treatment for Scaphicoma quadrifasciata
